# Symbiotic Fungi Alter the Acquisition of Phosphorus in *Camellia oleifera* through Regulating Root Architecture, Plant Phosphate Transporter Gene Expressions and Soil Phosphatase Activities

**DOI:** 10.3390/jof8080800

**Published:** 2022-07-29

**Authors:** Ming-Ao Cao, Rui-Cheng Liu, Zhi-Yan Xiao, Abeer Hashem, Elsayed Fathi Abd_Allah, Mashail Fahad Alsayed, Wiwiek Harsonowati, Qiang-Sheng Wu

**Affiliations:** 1 College of Horticulture and Gardening, Yangtze University, Jingzhou 434025, China; 202071717@yangtzeu.edu.cn (M.-A.C.); 202072809@yangtzeu.edu.cn (R.-C.L.); 2 Wuhan Forestry Workstation, Wuhan 430023, China; xzhyan@163.com; 3 Botany and Microbiology Department, College of Science, King Saud University, P.O. Box 2460, Riyadh 11451, Saudi Arabia; habeer@ksu.edu.sa (A.H.); malsayed@ksu.edu.sa (M.F.A.); 4 Plant Production Department, College of Food and Agricultural Sciences, King Saud University, P.O. Box 2460, Riyadh 11451, Saudi Arabia; eabdallah@ksu.edu.sa; 5 Agrobiology and Bioresources Department, School of Agriculture, Utsunomiya University, 350 Mine-Machi, Utsunomiya 321-8505, Japan; wiwiek_harsonowati@cc.utsunomiya-u.ac.jp

**Keywords:** mycorrhiza, oil crops, phosphatase, phosphate transporter, symbiosis

## Abstract

Plant roots can be colonized by many symbiotic fungi, whereas it is unclear whether and how symbiotic fungi including arbuscular mycorrhizal fungi and endophytic fungi promote phosphorus (P) uptake in *Camellia oleifera* plants. The objective of the present study was to analyze the effect of inoculation with a culturable endophytic fungus (*Piriformospora indica*), three arbuscular mycorrhizal fungi (*Funneliformis mosseae*, *Diversispora versiformis*, and *Rhizophagus intraradices*), and mixture of *F*. *mosseae*, *D*. *versiformis* and *R*. *intraradices* on plant growth, root architecture, soil Olsen-P, soil phosphatase activities, leaf and root P concentrations, and phosphate transporter gene expressions, in order to explore the potential and mechanism of these symbiotic fungi on P acquisition. All the symbiotic fungi colonized roots of *C*. *oleifera* after 16 weeks, with *P*. *indica* showing the best effect on fungal colonization. All the symbiotic fungi significantly increased acid, neutral, and total phosphatase activities in the soil, accompanied with an elevation of soil Olsen-P, of which *P*. *indica* presented the best effect. All symbiotic fungal treatments, except *D*. *versiformis*, significantly promoted plant growth, coupled with an increase in root total length, area, and volume. Symbiotic fungi almost up-regulated root *CoPHO1-3* expressions as well as leaf *CoPHO1-1*, *CoPHO1-3*, and *CoPHT1;4* expressions. Correlation analysis showed that P concentrations in leaves and roots were significantly positively correlated with root morphological variables (length, volume, and surface area) and soil acid, neutral and total phosphatase activities. It is concluded that symbiotic fungi, especially *P*. *indica*, played an important role in P uptake of *C*. *oleifera* plants through regulating root architecture, part plant phosphate transporter gene expressions and soil phosphatase activities.

## 1. Introduction

Phosphorus (P), one of macroelements in plants, is essential for plant growth [1,2]. There are two ways for plants to acquire P from the soil: one is directly absorbed by roots, and the other is absorbed by symbiotic fungi [3]. P in the soil is relatively rich, whereas it mainly exists in the form of bound P, which cannot be directly acquired by roots [4]. Phosphate (Pi) in the form of iron, aluminum and calcium in the soil further reduces the availability of soil P and seriously limits the acquisition of P by plants in the soil [1,3,5].

Symbiotic fungi promote the mineralization rate of organic P and the acquisition of P by plants [6,7]. In P deficient soils, the symbiosis of plants plays an indispensable role in the acquisition of soil P by plants [8,9]. Among symbiotic fungi, endophytic fungi are widely present in healthy tissues of living plants and can form a symbiotic relationship with host plants, thus triggering a positive response [10]. *Piriformospora indica* is an endophytic fungus isolated from the rhizosphere of desert plants in India that can be cultured on potato dextrose agar without roots [7]. Soil arbuscular mycorrhizal fungi (AMF) from the Glomeromycotina, are able to colonize roots of 72% of vascular plants to establish reciprocal arbuscular mycorrhizal symbiosis [11]. *P*. *indica* and AMF can be beneficial for plant growth, nutrient absorption, stress resistance, and disease resistance [7,12], especially in promoting P uptake by plants from the soil [13,14,15,16]. 

Plants usually adapt to soil P deficiency by altering root architecture, increasing the secretion of organic anions and phosphatases, and enhancing the expression of *phosphate transporter* (*PT*) genes [17]. *PTs* mediate P mobilization and uptake from the soil and its translocation and redistribution in root organelles, where PHT1 and PHO1, two important PTs in the roots, respond to P uptake from the soil as well as P loading from the xylem, respectively [18]. *PHT1* is the phosphorus transporter gene in plants, and *PHR1* binds to cis-acting element *P1BS* in the *PHT1* promoter to induce *PHT1* gene expression under the condition of P deficiency [2]. *PHO1* is responsible for transporting Pi to xylem exoplasmic space [19]. 

Yang et al. [7] revealed the increase in both soil phosphatase activities and plant P concentrations by trifoliate orange after inoculation with *P*. *indica*. Sahodaran et al. [20] found that an arbuscular mycorrhizal fungus, *Funneliformis mosseae*, improved nutrient utilization rate in nutrient-deprived soils, increased soil Olsen-P concentration, and thus promoted banana growth. Campos et al. [21] reported that *Rhizophagus intraradices* promoted the organic acid concentration in rhizosphere and root growth of *Triticum aestivum*, resulting in an increase in plant P concentrations. In mango plants, AMF dramatically promoted acid and alkaline phosphatase activities and P uptake in roots, thus promoting biomass production [22]. Therefore, symbiotic fungi play an important role in regulating plant P acquisition, which is involved in changes in soil physicochemical properties, root architecture, and *PT* gene expression. Despite the positive effect of AMF on plant P acquisition, there are AMF type differences in the promotion of plant P acquisition [1]. Studies in banana found that native AMF strains were more advantageous to banana plants than exotic AMF strains [20]. Therefore, there is a strong need to carry out functional evaluation of AMF for a particular plant to support future field applications. However, *P*. *indica* can be cultured in vitro, on a large scale, and has similar functions to AMF on P acquisition [7]. Whether there are differences between AMF and endophytic fungus *P*. *indica* on P acquisition is unclear.

*Camellia oleifera* Abel. is a small evergreen tree and can absorb a variety of harmful gases and dust, and it also beautifies the surrounding environment. The soil where *C*. *oleifera* grows is poor, especially with a severe P deficit [23]. Liu et al. [24] conducted high-throughput sequencing on roots and soils of *C*. *oleifera* in Wuhan, and found a total of 138 AMF species belonging to 10 genera in roots, indicating that the population of AMF in the rhizosphere of *C*. *oleifera* was rich. Wang et al. [25] also reported the increase in P concentrations in *C*. *oleifera* inoculated with *Glomus versiforme* and *G*. *mosseae*. Wu et al. [26] observed that *F*. *mosseae* inoculation enhanced the mineralization of soil bound P in *C*. *oleifera*. Whether the *in vitro* cultured endophytic fungus *P*. *indica* has a positive effect on P absorption of *C*. *oleifera* is unknown. 

The objective of this study was to analyze the effects of *P*. *indica* (PI), *F*. *mosseae* (FM), *Diversispora versiformis* (DV), *R*. *intraradices* (RI), and mixed-AMF of FM, DV, and RI on P concentration, root architecture, soil nutrients, soil phosphatase activity, and *PT* gene expression in *C*. *oleifera* plants, in order to explore the mechanism and application potential of symbiotic fungi in P acquisition of *C*. *oleifera* plants.

## 2. Materials and Methods

### 2.1. Symbiotic Fungal Inoculums

Based on the results of Wang et al. [25] and Wu et al. [26], three AMF species including FM (formely known as *G*. *mosseae*), DV (formely known as *G*. *versiforme*), and RI (formely known as *G*. *intraradices*), were used in the experiment. These AMF species were from the Institute of Root Biology, Yangtze University (Jingzhou, China) and propagated for 3 months by *Trifolium repens* L. under potted conditions. AMF-colonized roots and growth substrates were collected as AMF inoculums. Mixed-AMF was composed of FM, DV, and RI strains at a volume ratio of 1:1:1. The origin and propagation of PI were described by Yang et al. [7]. After suspension culture, spore suspension was used as the inoculant with a concentration of 2.173 × 10^7^ colony forming units/mL. 

### 2.2. Plant Culture

The seed of *C*. *oleifera* cv Changlin No. 40 was provided by Jingmen Tianyulang Agricultural Development Co., Ltd., Jingmen, China, and it was placed in autoclaved sands at room temperature. On 4 May 2021, two-leaf-old *C*. *oleifera* seedlings were transplanted into a plastic pot (12 cm × 11 cm × 15 cm) with 1.5 kg autoclaved substrate of sands and soils in a volume ratio of 1:3. Meanwhile, 80 g of mycorrhizal inoculums containing 1200−1400 spores were applied into the pot as the inoculated treatment; a total of 200 mL spore suspension of PI was applied into the pot as the PI treatment, based on the results of Yang et al. [7]. The same amount of autoclaved (121 °C, 0.1 MPa, 1 h) fungal inoculums (80 g AM fungal inoculum and 200 mL spore suspension of PI) were implemented as the control treatment. The soil physical and chemical properties were as follows: 103.88 ± 17.46 mg/kg of NH_4_-N concentration, 120.20 ± 16.23 of NO_3_-N concentration, 107.89 ± 10.36 mg/kg of Olsen-P concentration, and 97.41 ± 11.30 mg/kg of available potassium. All the seedlings were grown in a greenhouse with light quantum density of 948 μmol/m^2^/s, day and night temperature of 28 °C/23 °C, relative humidity of 70%, and 70% of maximum field water capacity. The experiment began in May 2021 and ended in September 2021 for 16 weeks. 

### 2.3. Experimental Design

The experiment was carried out using a single-factor design with six inoculated treatments: PI, FM, DV, RI, mixed-AMF, and non-fungi control. Each treatment had six replicates, along with two seedlings per replicate and a seedling per pot, in a total of 72 pots in a randomised arrangement.

### 2.4. Determinations of Variables

After 16 weeks of fungal inoculations, plant height was measured with a tape measure, stem diameter was measured with a vernier caliper, and number of leaves was counted. The plant was gently shaken off the soil attached to the root as the rhizosphere soil, and the shoot + root biomass was weighed, immediately treated with liquid nitrogen, and stored at −80 °C. The concentration of soil Olsen-P was determined by a Soil Nutrient Tachymeter (HM-TYA, Weifang, China) according to the user manual. 

The roots (six roots per treatment) were scanned by a scanner (J221A, Epson, Jakarta, Indonesia), and root architecture (total length, surface area, average diameter, and volume) was analyzed using WinRHIZO software (Regent Instruments Inc, Quebec, Canada). 

The fine roots were cut into about 1.0-cm-long root segments and stained with 0.05% trypan blue as outlined by Phillips and Hayman [27]. Fungal colonization was observed under a biological microscope (NE610, Ningbo, China). Fungal colonization rate (%) was estimated according to the formula described by Yang et al. [7]. 

P concentrations in roots and leaves were determined using an ICP-OES spectrometer (IRIS Advantage, Thermo, Waltham, MA, USA) after digested with nitric acid-perchloric acid. Acid, neutral, and alkaline phosphatases in the air-dried soil were extracted by acetate buffer (pH 5.0), citric acid-phosphoric acid buffer (pH 7.0), and boric acid buffer (pH 10.0), respectively, and the corresponding activities were assayed by the phenyl disodium phosphate method outlined by Wu et al. [28]. 

Six *PT* genes of *C*. *oleifera* were obtained from the NCBI database: *CoPHT1;1* (JX403969), *CoPHT1;2* (JX412956), *CoPHT1;3* (KF989483), *CoPHT1;4* (KF989484), *CoPHO1-1* (KU161157), and *CoPHO1-3* (KU161156). Primer Premier 5.0 software was utilized to design primer sequences of selected genes (Table 1). Frozen samples of roots and leaves were ground in liquid nitrogen, and total RNA was extracted using an EASY Spin Plus Plant RNA Kit (Aidlab). The PrimeScriptTM RT Reagent Kit with gDNA Eraser (Takara) was used for RNA reverse transcription. Real-time fluorescence quantitative expression analysis was done by a fluorescent dye method. The relative gene expression was calculated by a 2-^−ΔΔCt^ method [29], in which *EF-1α* was selected as the reference gene, and the data were normalized to the expressions of non-fungi control plants.

### 2.5. Statistical Analysis

The data obtained here were analyzed with the analysis of variance by the SAS software, and the significant difference between treatments was carried out using the Duncan’s multiple range test at the 5% level. The Pearson’s correlation coefficients were analyzed using the SAS software.

## 3. Results

### 3.1. Changes in Fungal Colonization

After 16 weeks of fungal inoculation, no fungal colonization was observed in roots of *C*. *oleifera* inoculated with non-fungi, while the inoculated plants represented 27% to 75% of root fungal colonization degree. Among them, inoculation with PI had the highest fungal colonization, reaching 75%, followed by RI, mixed-AMF, FM, and DV (Table 2).

### 3.2. Growth Responses under Different Fungal Inoculations

**Figure 1:**
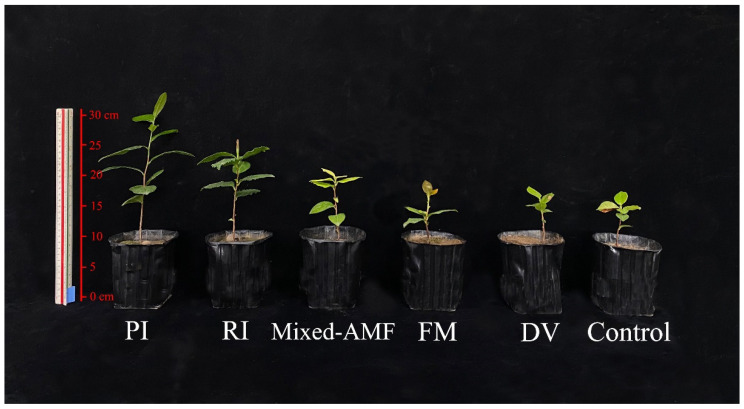
Changes in plant growth of *Camellia oleifera* plants after inoculation with symbiotic fungi. The abbreviation is the same as Table 2.

Compared with the control, DV treatment had no significant effect on plant growth parameters, while the other inoculations collectively improved plant height, stem diameter, leaf number, and plant (shoot + root) biomass to varying degrees (Figure 1; Table 2). Compared with non-fungal inoculation, plant height, stem diameter, leaf number, and plant (shoot + root) biomass were increased after symbiotic fungal inoculation: 103.1%, 96.8%, 129.2%, and 127.4% higher under PI inoculation; 46.6%, 42.7%, 60.4%, and 38.3% higher under RI conditions; 32.8%, 42.2%, 39.6% and 23.0% higher under mixed-AMF inoculation conditions. FM inoculation also promoted plant height, stem diameter, and leaf number by 24.2%, 35.1% and 35.4%, respectively, while it had no significant effect on plant (shoot + root) biomass, relative to non-fungal control. Overall, PI inoculation showed the best effects on growth promotion among the five symbiotic fungal treatments, while DV inoculation had no significant effect on plant growth.

### 3.3. Root Architecture Responses under Different Fungal Inoculations

**Figure 2:**
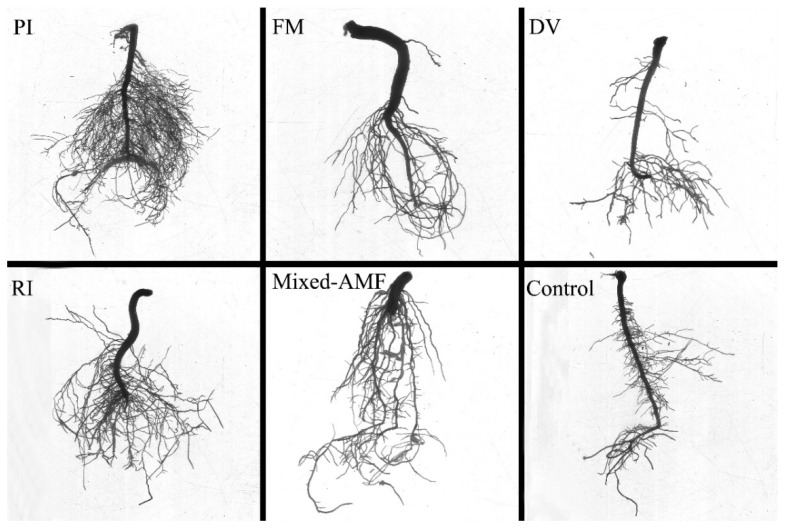
Changes in root architecture of *Camellia oleifera* plants after inoculation with symbiotic fungi. The abbreviation is the same as Table 2.

Root architecture is an important indicator in the amount of P intercepted by roots. Different symbiotic fungi exhibited different changes in root architecture (Figure 2; Table 3). Compared with the control, PI, FM, RI and mixed-AMF inoculations promoted root architecture to varying degrees, while DV inoculation had no significant effect (Table 3). The root length, surface area, and volume of inoculated seedlings were increased by 155.7%, 63.0%, and 284.0% under PI inoculation conditions and 141.3%, 55.3%, and 152.0% under RI-inoculation, respectively. Similarly, mixed-AMF significantly increased root length, surface area, and volume by 37.2%, 37.7%, and 36.0%, respectively, while FM only increased surface area and volume by 24.6% and 60.0%, respectively. In addition, compared with the control, inoculation with PI, FM, RI and mixed-AMF substantially reduced average diameter by 30.3%, 19.10%, 28.1%, and 23.6%, respectively.

### 3.4. Soil Olsen-P Responses under Different Fungal Inoculations

**Figure 3:**
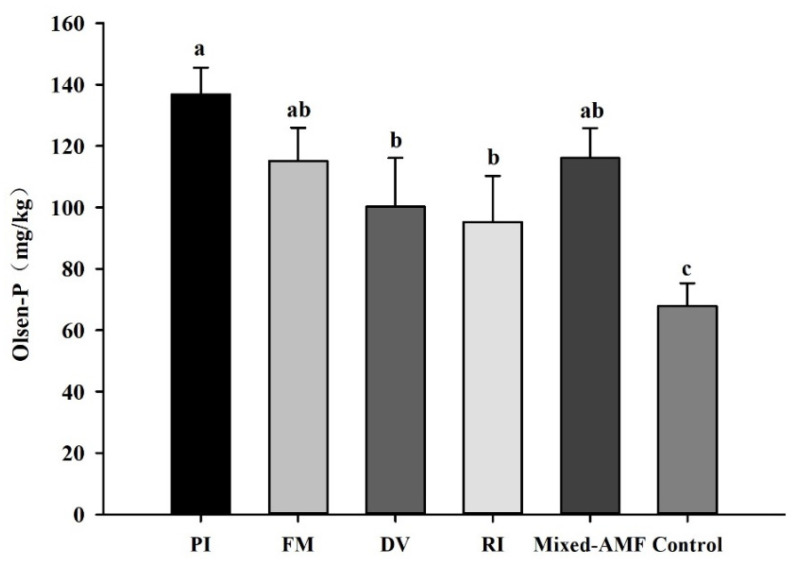
Effects of symbiotic fungi on soil Olsen-P concentrations of *Camellia oleifera* plants. Data (means ± SD, *n* = 6) followed by different letters above the bars indicate significant (*p* < 0.05) differences. The abbreviation is the same as Table 2.

All fungal inoculation treatments significantly increased soil Olsen-P concentrations. Compared with the control, soil Olsen-P concentrations in rhizosphere of PI-, FM-, DV-, RI- and mixed-AMF-inoculated seedlings increased by 101.5%, 69.6%, 47.8%, 40.4% and 71.0%, respectively (Figure 3).

### 3.5. Soil Phosphatase Responses under Different Fungal Inoculations

**Figure 4:**
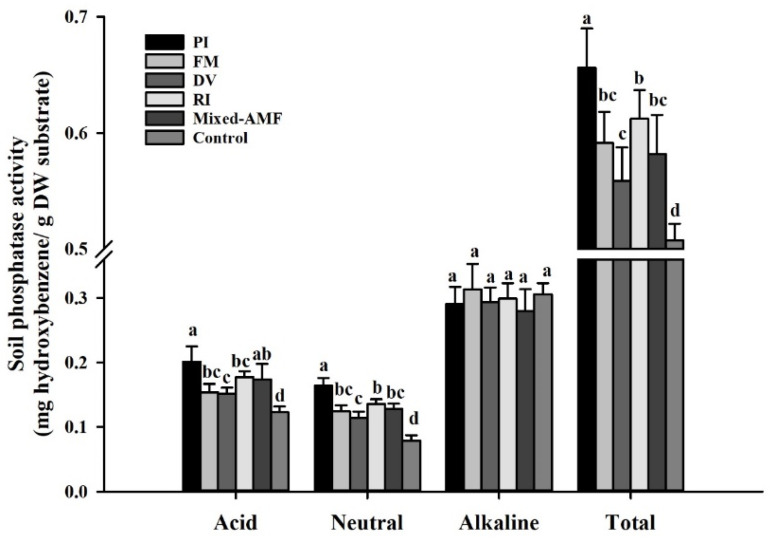
Effects of symbiotic fungi on soil phosphatase activities of *Camellia oleifera* plants. Data (means ± SD, *n* = 6) followed by different letters above the bars indicate significant (*p* < 0.05) differences. The abbreviation is the same as Table 2.

Symbiotic fungi promoted the activities of acid, neutral, and total phosphatase in rhizosphere of *C*. *oleifera* to varying degrees, while alkaline phosphatase activities in the soil of different treatments showed no significant difference (Figure 4). Compared with the control, inoculation with PI, FM, DV, RI, and mixed-AMF elevated soil acid phosphatase activity by 63.4%, 25.2%, 23.2%, 44.3%, and 41.5%, neutral phosphatase activities by 108.3%, 57.8%, 44.4%, 72.1%, and 62.2%, and total phosphatase activities by 29.3%, 16.5%, 10.1%, 20.6% and 14.6%, respectively. In general, PI treatment had the best promoting effect on soil phosphatase activity, followed by RI treatment.

### 3.6. Plant P Responses under Different Fungal Inoculations

**Figure 5:**
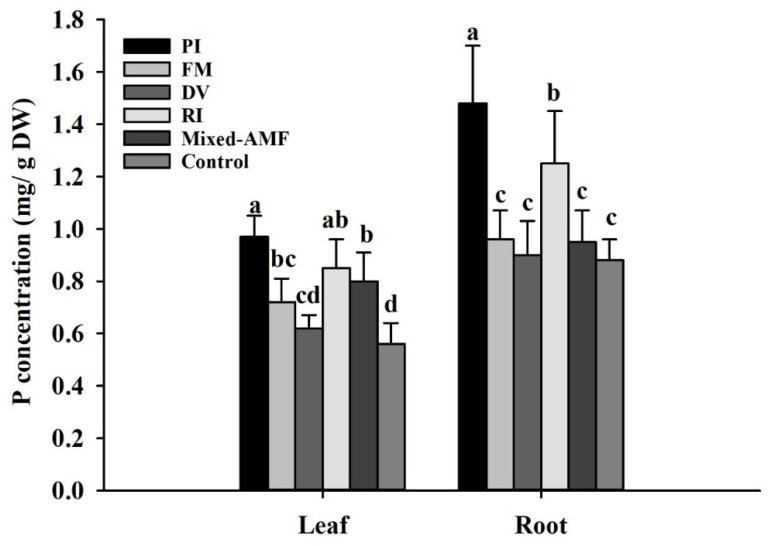
Effects of symbiotic fungi on leaf and root P concentrations of *Camellia oleifera* plants. Data (means ± SD, *n* = 6) followed by different letters above the bars indicate significant (*p* < 0.05) differences. The abbreviation is the same as Table 2.

Symbiotic fungi showed different effects on P acquisition in leaves and roots (Figure 5). Compared with the control, DV treatment has no significant effect on P concentration in leaves and roots, while PI, FM, RI and mixed-AMF significantly promoted P concentration in leaves by 73.2%, 28.6%, 51.8%, and 42.9%, respectively. In roots, only PI and RI promoted P concentration by 68.2% and 48.0%, respectively, with no significant difference between FM and mixed-AMF and the control.

### 3.7. PT Gene Expression Responses under Different Fungal Inoculations

**Figure 6:**
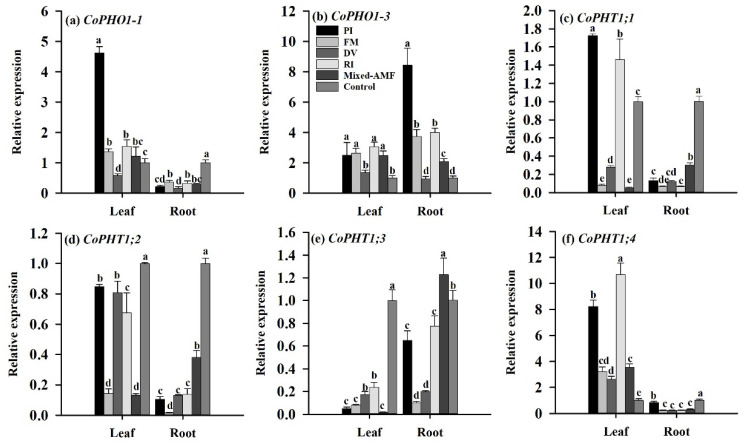
Effects of symbiotic fungi on expressions of genes in leaves and roots of *Camellia oleifera* plants. Data (means ± SD, *n* = 3) followed by different letters above the bars indicate significant (*p* < 0.05) differences. The abbreviation is the same as Figure 1.

There were differences in the expression of *PT* genes in leaves after inoculation with symbiotic fungi (Figure 6a–f). PI and RI inoculations significantly induced *CoPHO1-1*, *CoPHO1-3*, *CoPHT1;1* and *CoPHT1;4* gene expressions in leaves by 4.12-fold, 2.50-fold, 1.73-fold, and 8.23-fold under PI and by 1.55-fold 3.06-fold, 1.46-fold, and 10.68-fold under RI, respectively. FM up-regulated leaf *CoPHO1-1*, *CoPHO1-3*, and *CoPHT1;4* gene expressions by 1.36-fold, 2.64-fold, and 3.23-fold, and mixed-AMF increased the expression of these genes by 1.22-fold, 2.48-fold and 3.54-fold, respectively. DV only induced a 2.62-fold up-regulation of leaf *CoPHT1;4* expressions (Figure 6f), along with the inhibited expression of *CoPHO1-1* in leaves (Figure 6a). All inoculations inhibited *CoPHT1;2* and *CoPHT1;3* expressions in leaves (Figure 6d,e), compared to the control.

Compared with the control, all the symbiotic fungal inoculations significantly down-regulated expressions of *CoPHO1-1*, *CoPHT1;1*, *CoPHT1;2*, and *CoPHT1;4* genes in roots (Figure 6). Only mixed-AMF up-regulated *CoPHT1;3* expressions in roots, along with down-regulated expressions of *CoPHT1;3* between the other fungal inoculations and the control (Figure 6e). All symbiotic fungi, except DV, induced up-expression of *CoPHO1-3* gene in roots, with PI expression being the highest at 8.43-fold (Figure 6b).

### 3.8. Correlation Analysis

Correlation studies showed that root fungal colonization was significantly and positively correlated with leaf P concentration, but not root P concentration (Table 4). In addition, leaf and root P concentration was significantly and positively correlated with root total length, surface area, and volume, but root P concentration was negatively correlated with root average diameter. Leaf and root P concentration was also significantly and positively correlated soil acid, neutral, and total phosphatase activities.

## 4. Discussion

P can be acquired by symbiotic fungi [19,20]. In our study, all the symbiotic fungi could colonize roots of *C. oleifera*, where PI had the highest colonization rate (75%), and DV had the lowest colonization rate (27%). The symbiosis between symbiotic fungi and plants requires the plant to provide carbohydrates to the fungi for growth maintenance [30]. The change in plant growth in PI- and DV-inoculated plants represented that the fungal colonization was associated with symbiotic fungi-improved growth responses [31]. In the present study, PI, FM, RI and mixed-AMF dramatically promoted plant growth parameters to varying degrees, which was consistent with the findings of Standish et al. [32] in mango. However, the DV treatment in our study had no significant effect on plant growth performance, which may be related to the low colonization level of DV in roots. In addition, the PI treatment in our study had the best effect on plant growth promotion, indicating that this fungus has good compatibility with *C. oleifera*. All the fungal inoculations significantly promoted leaf P concentration, and PI and RI treatments also promoted root P concentration. Plant growth improvement was consistent with the trend of fungi-induced plant P acquisition, indicating that symbiotic fungi promoted P acquisition of host plants, thus resulting in improved growth [31].

P has low mobility in the soil, and thus great roots can help roots absorb more P [21,33]. In this study, PI, FM, RI, and mixed-AMF collectively promoted root length, surface area, and volume of *C. oleifera* to a certain extent, which is in accordance with the previous findings of Zhang et al. [34] in citrus, indicating that symbiotic fungi promote P uptake in plants by improved root architecture. PI and RI treatment had a strong promoting effect on root architecture, while DV treatment showed no effect on root architecture, suggesting that there was a specificity in the interaction between symbiotic fungi and *C. oleifera* plants [35]. P concentrations of leaves and roots were significantly positively correlated with root length, surface area, and volume, further suggesting that the symbiotic fungi-improved root architecture is an important reason for promoting P acquisition of host plants [18]. Such root changes may be associated with auxin levels and soil properties induced by symbiotic fungi [36,37]. Furthermore, many genes (e.g., ROOT APICAL MERISTEM) and transcription factors (e.g., SOMERERO) are involved in root architecture remodeling [18], and whether and how symbiotic fungi affect the transcription levels of these genes and transcription factors to improve root architecture, remains to be further investigated.

Soil phosphatases can stimulate the transformation of soil organic P to inorganic P, thus improving Olsen-P concentration in the rhizosphere for the P acquisition of roots [5]. Mycorrhizal fungi release phosphatase into rhizosphere to increase soil phosphatase activities and thus ultimately promote the mineralization of organic phosphorus [18,38]. Our study revealed that all the inoculation treatments significantly promoted soil Olsen-P concentration, which is consistent with the results of Shao et al. [37]. Among them, soil Olsen-P concentration in PI-inoculated plants was the highest, which may be linked to the higher soil acid, neutral, and total phosphatase activities induced by PI. In addition, P concentrations in roots and leaves were significantly positively correlated with soil acid, neutral and total phosphatase activity, indicating that the increase of soil phosphatase activity induced by symbiotic fungi plays an important role in the symbiotic fungi-induced P acquisition.

Our study indicated that all fungal inoculations inhibited root *CoPHO1-1*, *CoPHT1;1*, *CoPHT1;2*, *CoPHT1;3*, and *CoPHT1;4* expressions, along with an exception for the up-regulation expression of root *CoPHT1;3* after inoculation with mixed-AMF only. This is in agreement with the results reported by Shu et al. [39], in which mycorrhizal fungi induced down-regulation of *PtaPT1*, *PtaPT2*, *PtaPT3*, and *PtaPT7* in roots of *Poncirus trifoliata* under P deficit conditions. This suggested that in *C. oleifera*, the promotion of P uptake by these symbiotic fungi is not entirely dependent on fungi-induced *PTs* expressions, but is associated with the improvement of root architecture and the increase of soil phosphatase activity. In addition, higher soil Olsen-P concentrations in the rhizosphere of fungi-inoculated plants may also be a reason of down-regulation of these genes. All the fungal treatments, except DV, induced up-regulation of *CoPHO1-3* expression in leaves and roots, along with the corresponding increase of P in these tissues. PHO1 is involved in the xylem loading of P [18]. The results suggest that *CoPHO1-3* gene plays a key role in the redistribution of P from roots to leaves of mycorrhizal *C. oleifera* plants, while more work needs to be done around *CoPHO1-3*.

In addition, both PI and RI inoculations up-regulated *CoPHO1-1*, *CoPHO1-3*, *CoPHT1;1*, and *CoPHT1;4* expressions in leaves, with *CoPHT1;4* expressions up-regulated up to 8.23- and 10.68-fold, respectively, compared with uninoculation treatment. FM and mixed-AMF also induced expressions of *CoPHO1-1*, *CoPHO1-3*, and *CoPHT1;4* in leaves, while the magnitude of the up-regulation of these genes was less than that under PI and RI inoculations conditions, indicating that *CoPHT1;4* may have a key role in symbiotic fungi-induced P uptake in leaves. In a word, symbiotic fungus-induced root *CoPHO1-3* gene and leaf *CoPHT1;4* gene have a key role in P uptake of *C. oleifera.*

## 5. Conclusions

In brief, P absorption of *C. oleifera* was differentially regulated by symbiotic fungi inoculation, among which PI had the best effect on increasing leaf (73.2%) and root (68.2%) P concentration, with no significant effect of DV. Part of symbiotic fungi used here accelerated plant growth performance, improved root architecture, increased soil acid and neutral phosphatase activities, and induced expressions of root *CoPHO1-3* gene and leaf *CoPHT1;4* gene, so as to promote the uptake of P in *C. oleifera* plants. These pathways are interrelated and influence each other, and it is not clear which of them is responsible for the main function. Among all the symbiotic fungi used here, PI showed the best effect on plant growth and P acquisition, and thus culturable PI can be used as the bio-fertilizer in *C. oleifera*. Among the AMF species used here, RI also showed relatively good effects, although weaker than PI, but higher than other AMF, which can be properly considered in *C. oleifera*.

## Figures and Tables

**Table 1:** The primer sequence of genes in qRT-PCR.

Gene Names	Accessions	Sequences (5′→3′)
*CoPHT1;1*	JX403969	F: GTTCTTGGCGGAGTCAATTTC
		R: CATCCTCATCTTCCTCGTTCTC
*CoPHT1;2*	JX412956	F: TCCCTTTGCTTCTTCCGATTT
		R: CGAGTCCTCTTGTTGGCATATT
*CoPHT1;3*	KF989483	F: GAGTCAGAGCAGCAGAAAGTAG
		R: TGTAGTCCCAAGCAAGTGAAG
*CoPHT1;4*	KF989484	F: CCGTTACACCGCCCTTATC
		R: CTGGGTTCTTCAGCCATCTT
*CoPHO1-1*	KU161157	F: AGCAGCCCTTGAAGTCATTAG
		R: GAACTTGCCCGCATTGTTTAG
*CoPHO1-3*	KU161156	F: GAGCTTTCAGTGGCCTAACA
		R: GTCGCCTCACCGAGTTTATC
*EF-1α*	KC337050	F: AGACTGTGGCTGTTGGTGTT
		R: ATCCAAACCCGCACAGTTCA

**Table 2:** Effects of symbiotic fungi on plant growth responses of *Camellia oleifera* plants.

Treatments	Fungal Colonization Rate (%)	Height (cm)	Stem Diameter (mm)	Leaf Number (#/Plant)	Plant (Shoot + Root) Biomass (g/plant)
PI	75 ± 7 a	19.50 ± 3.70 a	3.64 ± 0.40 a	11.0 ± 1.1 a	5.23 ± 0.55 a
FM	54 ± 5 c	11.92 ± 0.95 b	2.50 ± 0.68 bc	6.5 ± 1.0 b	2.71 ± 0.38 bc
DV	27 ± 4 d	9.50 ± 1.03 c	1.94 ± 0.46 cd	5.0 ± 0.9 c	2.19 ± 0.29 d
RI	67 ± 7 ab	14.07 ± 1.79 b	2.64 ± 0.43 b	7.7 ± 1.6 b	3.18 ± 0.39 b
Mixed-AMF	60 ± 8 b	12.75 ± 1.17 b	2.63 ± 0.65 b	6.7 ± 1.2 b	2.83 ± 0.40 b
Control	0 ± 0 e	9.60 ± 0.74 c	1.85 ± 0.15 d	4.8 ± 0.7 c	2.30 ± 0.23 cd

Data (means ± SD, *n* = 6) followed by different letters in the column indicate significant (*p* < 0.05) differences. Abbreviation: AMF, arbuscular mycorrhizal fungi; Control, uninoculation with any fungi; DV, *Diversispora versiformis*; FM, *Funneliformis mosseae*; PI, *Piriformospora indica*; RI, *Rhizophagus intraradices*; mixed-AMF, mixture of *D*. *versiformis*, *F*. *mosseae* and *R*. *intraradices*.

**Table 3:** Effects of symbiotic fungi on root morphological parameters of *Camellia oleifera* plants.

Treatments	Length (cm)	Surface Area (cm^2^)	Average Diameter (mm)	Volume (cm^3^)
PI	138.76 ± 14.80 a	13.76 ± 1.35 a	0.62 ± 0.05 b	0.96 ± 0.12 a
FM	65.50 ± 8.33 bc	10.52 ± 0.65 b	0.72 ± 0.11 b	0.40 ± 0.04 c
DV	41.38 ± 4.78 d	7.51 ± 1.13 c	0.95 ± 0.10 a	0.23 ± 0.03 d
RI	130.93 ± 17.80 a	13.11 ± 1.05 a	0.64 ± 0.07 b	0.63 ± 0.07 b
Mixed-AMF	74.44 ± 8.02 b	11.62 ± 0.88 b	0.68 ± 0.04 b	0.34 ± 0.02 c
Control	54.26 ± 6.15 cd	8.44 ± 0.82 c	0.89 ± 0.07 a	0.25 ± 0.03 d

Data (means ± SD, *n* = 6) followed by different letters in the column indicate significant (*p* < 0.05) differences. The abbreviation is the same as Table 2.

**Table 4:** Correlationships between leaf and root P concentrations and root fungal colonization rate, root morphological variables, and soil phosphatase activities (*n* = 24).

	Fungal Colonization	Root Architecture				Soil Phosphatase			
		Total Length	Surface Area	Average Diameter	Volume	Acid	Neutral	Alkaline	Total
Leaf P	0.94 **	0.91 **	0.95 **	−0.74	0.91 *	0.97 **	0.95 **	−0.42	0.95 **
Root P	0.73	0.95 **	0.85 *	−0.91 *	0.99 **	0.85 *	0.84 *	−0.21	0.87 *

* *p* < 0.05; ** *p* < 0.01.

## Data Availability

All the data supporting the findings of this study are included in this article.
